# Natural Remedies for the Treatment of Beta-Thalassemia and Sickle Cell Anemia—Current Status and Perspectives in Fetal Hemoglobin Reactivation

**DOI:** 10.1155/2014/123257

**Published:** 2014-10-02

**Authors:** Noel Yat Hey Ng, Chun Hay Ko

**Affiliations:** ^1^School of Life Sciences, The Chinese University of Hong Kong, Shatin, New Territories, Hong Kong; ^2^Institute of Chinese Medicine, The Chinese University of Hong Kong, Shatin, New Territories, Hong Kong; ^3^CUHK Shenzhen Research Institute, The Chinese University of Hong Kong, Shenzhen, Hong Kong

## Abstract

For the treatment of *β*-thalassemia and sickle cell disease (SCD), pharmacological induction of fetal hemoglobin (HbF) production may be a promising approach. To date, numerous studies have been done on identifying the novel HbF-inducing agents and understanding the underlying mechanism for stimulating the HbF production. In this review, we have summarized the identified HbF-inducing agents by far. By examining the action mechanisms of the HbF-inducing agents, various studies have suggested that despite the ability of stimulating HbF production, the chemotherapeutic agents could not be practically applied for treating *β*-hemoglobinopathies, especially *β*-thalassemia, due to the their cytotoxicity and growth-inhibitory effect. Owing to this therapeutic obstacle, much effort has been put on identifying new HbF-inducing agents from the natural world with the combination of efficacy, safety, and ease of use. Therefore, this review aims to (i) reveal the novel screening platforms for identifying potential inducers with high efficiency and accuracy and to (ii) summarize the new identified natural remedies for stimulating HbF production. Hopefully, this review can provide a new insight into the current status and future perspectives in fetal hemoglobin reactivation for treating *β*-thalassaemia and SCD.

## 1. Introduction

Genetic disorders caused by mutations in the *β*-globin gene are widely known as the human *β*-hemoglobinopathies, in which *β*-thalassemia and sickle cell disease (SCD) are the most prevalent ones, particularly in the Mediterranean, Africa, and Southeast Asia, leading to great mortality and morbidity [1–4]. The high occurrence of the *β*-thalassemia and SCD mutations is due to the reason that both cause mild severity of malarial infection in the heterozygous state [5–7]. However, in the homozygous state, these mutations shorten the lives of affected ones [5].

*β*-thalassemia is caused by the inherited mutations in the *β*-globin gene complex, resulting a total absence or severe decrease in the production of *β*-globin chains [8, 9]. The lack of *β*-globin chain production leads to the accumulations and precipitations of free intracellular α-globin chains, which may consequently result in premature hemolysis of red blood cells and apoptosis of erythroid precursor [8, 10, 11]. Ineffective erythropoiesis has also been known to be related to inefficient iron utilization [12]. Therefore, the combining effects of ineffective erythropoiesis, hemolysis, and hypersplenism are the main culprit of severe anemia in *β*-thalassemia patients [5].

SCD is an inheritable autosomal recessive genetic blood disorder. It is characterized by the abnormal appearance of the red blood cells which are rigid and sickled. SCD is attributed to a point mutation at the coding sequence of the *β*-globin gene which causes the substitution of glutamate by valine in the glutamic acid at the sixth position of *β*-globin protein, and thus forming a sickle hemoglobin (HbS, *α*_2_ *β*_2_^S^) when incorporating into a hemoglobin tetramer [13, 14]. HbS will polymerize inside the red blood cells under hypoxic condition, resulting in the alternation of the shape of red blood cells as well as their function.

Currently, the clinical manifestation in *β*-hemoglobinopathies is blood transfusion and gene transfer therapy. However, long-term transfusion therapy may cause iron overload in patients from the gradual breakdown of transfused blood which may eventually result in cardiac failure and/or even death [5]. Though the advance in iron chelation can help to remove excess iron in patients, the survival rate is greatly dependent on the iron chelation regimens [15]. Allogeneic hematopoietic stem cell transplantation (HSCT) is one of the gene transfer therapies aimed at the underlying molecular causes of SCD and *β*-hemoglobinopathies. Several hundred SCD and thalassemia patients have successfully experienced HSCT with promising results [16, 17]. Nevertheless, there is a great likelihood that HSCT will be limited to a small proportion of hemoglobinopathy patients as evidence has shown merely younger patients and those who have not developed significant disease complications have gained the best results in HSCT [18]. Also, most successful transplantations have to utilize stem cells from matched sibling donors making HSCT a challenging therapy for some patients [16]. Therefore, HSCT may not be applicable to many current patients. Transferring of *γ*- or *β*-hematopoietic stem cells of patients can be another therapy option for *β*-thalassemia patients [5]. It has taken a long period of time to have the clinical gene transfer protocol being approved since the transduction of human hematopoietic stem cells and gene expression must reach certain efficiency and high level [19]. Despite the fact that this approach has successfully passed its initial human trial, previous studies reported the issues regarding low autoploidy recombination [20], insertional mutagenesis, and effect of inserted vectors on the expression of nearby genes could possibly limit the application [21, 22].

Apart from gene therapy, fetal hemoglobin reactivation by chemical agents appears promising enough to develop into effective interventions to cure human *β*-hemoglobinopathies. Previous studies have revealed that homozygous *β*-thalassemia patients will not suffer severe anemia until fetal *γ*-globin genes are silenced and that patients carrying hereditary persistence of fetal hemoglobin (HPFH), meaning fetal hemoglobin (HbF) is abnormally persisted at high level in adults, will only suffer mild anemia [8, 13, 23–27]. More evidences also supported that HPFH can improve the severity of both *β*-thalassemia and SCD [1]. Therefore, it have been suggested that increasing the synthesis of fetal hemoglobin (HbF) by reactivating fetal *γ*-globin gene can be a potential therapy in patients suffering *β*-thalassemia or SCD. It is expected that the pharmacological induction of HbF can correct the globin chain imbalance in *β*-thalassemia patients, while inhibit HbS polymerization in SCD patients [28–32].

In recent years, much effort has been made to identify the naturally occurring inducers and drug treatments which can increase the synthesis of HbF and promote the expression of fetal *γ*-globin gene. Some chemotherapeutic agents, for example, 5-azacytidine and hydroxyurea, (HU) have been reported due to their ability to enhance HbF production [31, 33, 34]. Yet, most of these currently identified HbF-inducing agents exhibit low efficacy and specificity, myelotoxicity, and carcinogenesis as well as modest responses to treatment which greatly limit their usefulness in the clinical practice [5, 35, 36]. Owing to this, (i) discovering novel screening platform for identifying potential inducers with high efficiency and accuracy and (ii) identifying new HbF-inducing agents from the natural world with the combination of efficacy, safety, and ease of use will be high on the agenda [5].

## 2. *In Vivo* and* In Vitro *Screening Platforms

With the aim of determining the therapeutic potency of the novel inducing compounds and studying the underlying regulatory mechanism of the embryonic and fetal human globin genes expression, various* in vitro* and* in vivo *screening platforms have been widely utilized. For* in vitro *models, there are six human cell lines carrying an embryonic-HbF phenotype; they are K562 human chronic myelogenous leukemia cells, M-TAT, NSMeg, OCIM1, OCMI2, and AS-E2, while K562 cell line is one of the most well-known and widely used screening platforms for HbF inducers [13, 30]. Another seven human cell lines which are capable of synthesizing both *γ*- and *β*-globin chains are JK-1, KMOE-2, KU812, LAMA-84, TF-1, TN922, and AP217; yet, KU812 cell line is comparatively unique and useful from the others as it can undergo a spontaneous differentiation which can be observed [30, 37–39]. Moreover, human bone marrow CD34+ hematopoietic progenitors drawn from *β*-thalassemia patients and primary erythroid progenitors stem cells (EPSCs) obtained from peripheral blood are also great* in vitro* models to study the effect of different HbF-inducing agents [1, 13].

Back to the 1980s, baboons, a nonhuman primate model, have already been used for the study of fetal and adult hemoglobin synthesis during fetal development [40, 41]. There was an influential research conducted by De Simone and colleagues that the adult baboon has been shown to respond to erythropoietic stress with the reverse hemoglobin switch during which an increase in the number of HbF-containing erythrocytes (F cells) and an increase in HbF synthesis can be observed in adult baboon [40, 42, 43]. However, this animal model is expensive to purchase, feed, and maintain in conditions appropriate to modern animal husbandry. In order to understand the influence of HbF synthesis and its induction mechanism, till the 90s, researchers have successfully discovered that transgenic mice carrying human A *γ*-globin gene, which can act as a new* in vivo *model for screening novel pharmaceutical compounds for HbF induction in the adult [44, 45].

## 3. Overviews on HbF Inducers

Up to date, researchers have discovered numerous HbF-inducing agents. These inducers can be categorized into several classes based on their mechanisms of action [13, 46] as listed in Table 1. Some of them are classified as chemotherapeutic agents, for example, hydroxyurea (HU), 5-azacytidine (5-Aza), decitabine, and citarabine. HU is also known as a ribonucleotide reductase inhibitor due to its ability of inhibiting DNA analysis, while 5-Aza, decitabine and citarabine are DNA methyltransferase inhibitors who responsible for the hypomethylation of DNA [8, 13]. Several short-chain fatty acids (SCFAs) specifically stimulate transcription in the *γ*-globin gene promoter through histone deacetylase HDAC inhibition, resulting in global histone hyperacetylation [5, 47]. In contrast, some studies argue that globin histone hyperacetylation induced is not the primary mechanism of SCFA [5]; yet, HDAC inhibitors are often potent *γ*-globin inducers [47, 48]. Rapamycin belongs to the family of mTOR inhibitors. In erythroid precursor cells, rapamycin preferentially induce *γ*-globin mRNA accumulation, while being only minor for *β*-globin and none for α-globin mRNAs [49]. As its HbF-inducing effect is not related to cytotoxicity and cell growth inhibition, scientists are very interested in further studying if the enhancement of *γ*-globin mRNA medicated by rapamycin is associated with XmnI polymorphism [49]. There are studies showing that K562 cells treated with DNA-binding agents, such as mithramycin, have led to erythroid differentiation and sharp enhancement of *γ*-globin mRNA level [50]. Through PCR arrested experiments, it is found that these DNA-binding drugs were capable of interacting with *γ*-globin promoter of human genomic DNA [50]. In recent years, researches have been done on immunomodulatory drugs, such as thalidomide, revlimid, and pomalidomide. Their exhibited HbF-inducing activity has been revealed in K562 or primary human erythroid cultures. Further study has demonstrated their activity is associated with the increase in histone acetylation at *γ*-globin gene promoter [51]. Erythropoietin (EPO) is a cytokine that have been shown to induce HbF production during* in vitro *differentiation of primary human cells in several trials [47, 52–55]. It also stimulates red blood cells production, prolongs the survival of erythroid cells, and decreases the incident of programmed cell death [47].

## 4. Natural Remedies as HbF Inducer

In recent years, scientists have conducted numerous studies in order to identify the natural remedies that could be possibly applied in treating *β*-hemoglobinopathies, including SCD and *β*-thalassemia, summarized in Table 2. The extract of* Aegle Marmelos* containing bergatene was found to be responsible for the activation of erythroid differentiation and HbF induction in human leukemic K562 cells [31, 69]. Citropten and bergatene are the active ingredients in bergamot juice. They are powerful inducers of erythroid differentiation, *γ*-globin gene expression and HbF synthesis in human erythroid cells. Thus, it is known as a potential therapeutic approach for both *β*-thalassemia and sickle cell anemia [69]. In addition, Nicosan (formerly known as Niprisan), an ethanol/water extract from Nigeria indigenous plants, has successfully demonstrated a significant anti-sickling effects* in vitro *as well as* in vivo* [70, 71].

Angelicin can be found in the fruit of* Angelica arcangelica*. There is evidence demonstrating that angelicin is a powerful inducer of erythroid differentiation, enhancement of the HbF synthesis in erythroid progenitors and *γ*-globin mRNA accumulation of human leukemia K562 cells [8, 73]. Red wine, especially the skin of grapes, contains resveratrol which mimics the HbF-inducing activity of HU [8]. Its function in increasing the *γ*-globin mRNA in human erythroid precursors has been confirmed [8]. Since *β*-thalassemia cells exhibit a high level of oxidative stress, which eventually shorten the survival of erythroid cells in *β*-thalassemia patients, resveratrol which exhibits both antioxidant activity and HbF inducing property can become a very promising HbF inducer from the natural world [74].

Rapamycin is isolated from* Streptomyces hygroscopicus,* a bacterial species being found in the soil of Easter Island. It has the ability to increase HbF production in cultures of erythroid precursors from *β*-thalassemia patients without cytotoxicity or growth-inhibitory effect [8, 13]. Apart from this, in* Streptomyces *species, mithramycin can be easily isolated. It is a DNA-binding drug which has the potential to induce *γ*-globin mRNA accumulation and HbF production in erythroid cells from healthy human subjects as well as *β*-thalassemia patients [75].

Recently, it is reported that an Indian almond, called* Terminalia catappa*, has long been used as a traditional herbal treatment for SCA in Nigeria [78]. Aimola et al. has then demonstrated* Terminalia catappa* distilled water active fraction (TCDWF) from* Terminalia catappa* leaves exhibit a stimulatory effect on the HbF production in primary erythroid prohenitor stem cells (EPSCs) [77].* Terminalia catappa* consists of flavonoids, alkaloids, and anthraquinones. Also, through gas chromatography-mass spectrometry (GCMS), it is shown that a group of highly related long-chain fatty acids, for example, hexadecanoic acid, 10-octadecenoic acid, and octadecenoic acid, are present in the TCDWF [77]. Yet, further investigation is required in order to confirm the biological activities of these active compounds present in the TCDWF.

For the Chinese herbal medicine, YiSui ShengXue Granule (YSSXG), a complex prescription made up of 11 different kinds of Chinese herbal medicines, has shown to be effective in enhancing the HbF expression and inhibiting ineffective hematopoiesis [76, 79]. Current research has further confirmed this complex prescription has the ability to increase *γ*-globin gene expression and alter the expression of genes that involved in the survival, proliferation, and terminal differentiation of erythroid cells [76]. Recently, some researchers found that cucurbitacin D (CuD) in a Chinese medicinal herb, called* Fructus trichosanthis *(FT), exhibits a higher potency in HbF induction compared with hydroxyurea since there is evidence showed CuD results in a higher fetal cell percentage and greater HbF content in K562 cells with much lower cytotoxicity [1].

## 5. Mechanism of Actions of HbF Induction

Up to date, more than 70 HbF-inducing agents have been described [5]. Scientists have started to put effort on appreciating their underlying molecular mechanisms as well as verifying their target molecules.

### 5.1. p38 MAPK Pathway

The first piece of evidence regarding the cell signaling of the HbF induction has suggested that, in K562 cells, butyrate induces erythroid differentiation and hemoglobin production through p38 MAPK pathway [1, 80]. Several years later, another group of scientists has interestingly revealed that, without drug treatment, the *γ*-globin mRNA level is increased sufficiently solely by overexpressing MAPKK3 and MAPKK 6 which are the direct upstream activators of p38 in K562 cells [81]. According to Mabaera et al., p38 MAPK signaling pathway is critical for the upregulation of the production of HbF [5]. Different environmental stresses could activate the p38 MAPK signal pathway which will subsequently cause apoptosis, cell growth and erythroid differentiation. Various studies have also revealed the effect of numerous HbF-inducing agents, such as butyrate [82], apicidin [83], and trichostatin A [84], are associated with p38 MAPK signaling pathway. Therefore, they both indicated p38 MAPK pathway plays a vital role in promoting *γ*-globin gene expression.

During the past few years, researchers come up with different mechanistic models of HbF induction while most of the models are generally based on what are thought to be the primary actions of HbF-inducing agents, for instance, global DNA hypomethylation induced by DNA methyltransferase inhibitors (DNMT inhibitors) or global histone hyperacetylation induced by histone deacetylase inhibitors (HDAC inhibitors), including SCFA derivatives. However, when we come across with the recent key experimental findings, including the fact that 5-Aza can promote HbF production without DNA hypomethylation, that *γ*-globin promoter hypomethylation is inadequate to stimulate gene expression, and that the ability of HDAC inhibitors to induce HbF is regardless of the potency of HDAC inhibitions, some of these proposed mechanistic models are no longer capable to explain those results. Therefore, it is of essence to propose a new model of HbF induction which is valid to most HbF-inducing agents and can adequately account for the recent experimental results [5].

Mabaera et al., through integrating recent results of cell-signaling experiments with the stress erythropoiesis model, have proposed a new model called the cell stress signaling model [5]. They suggested that the key effect of most HbF-inducing agents is to activate the cell stress signaling pathways during adult erythropoiesis which subsequently lead to *γ*-globin gene expression and HbF production. It is found that certain HbF-inducing agents, such as HU, Butyrate (SCFA), Thalidomide (IMiD), Trichostatin A (HDAC Inhibitor), and anisomycin, can activate the corresponding cell stress responses, including nitric oxide, oxidative stress (ROS), osmotic shock, and protein synthesis inhibition. These cell stress responses will eventually activate the downstream p38 MAPK signaling pathway, including downstream kinases and transcription factors, and thus result in *γ*-globin gene expression and HbF production. Besides p38 MAPK signaling pathway, the potential of cAMP signaling pathway in HbF production has also been mentioned in the cell stress signaling model. There are findings suggested that in primary erythroid cell cultures, cAMP response element binding protein (CREB) is activated by cAMP-activated protein kinase A instead of p38 MAPK pathway. The phosphorylated CREB will then activate the downstream transcription factors and eventually lead to *γ*-globin gene expression. Therefore, not only is the cell stress signaling model applicable to most of the HbF-inducing agents but also is able to explain the key findings of some of the previous experiments [5].

### 5.2. Roles of ERK and JNK

In the previous studies, researchers have found out that stimulating ERK signaling pathway leads to megakaryocytic differentiation; contrastingly, suppressing ERK signaling pathway results in the enhancement of the erythroid phenotype as well as the *γ*-globin mRNA expression [83]. Moreover, the evidence that ERK inhibitor U0126 has the ability to stimulate *γ*-globin gene expression and HbF production in human erythroid progenitor cells suggested the inhibition of ERK can possibly lead to the promotion of HbF production [84]. The involvement of JNK in erythroid differentiation still remained debatable [76, 85–87]. It is reported that pretreatment with HU in K562 cells has led to a significant inhibition of JNK [85]. Moreover, short chain fatty acid derivatives (SCFAD), such as butyrate and valproate, did not result in JNK phosphorylation; thus, there was not any significant changes on JNK pathway in K562 cells [76, 87]. Nevertheless, in mouse erythroleukemia cells, experimental findings have suggested that activation of JNK is crucial for erythropoietin-induced erythroid differentiation [86, 88]. As a result, whether JNK plays a significant role in HbF induction still remains to be investigated in the future.

### 5.3. Trans-Acting Factor and Cis-Acting Element of the Globin Gene

The potential of reactivating fetal hemoglobin via manipulating the gene transcription of *β*- or *γ*-globin has been gained much attention recently. There is escalated number of publications regarding the genetic regulatory mechanism of developmental stage-specific expression of *β*-globin genes. The mechanisms accounting for switching globin-genes expression during the development are highly regulated by cis-acting elements and trans-acting factors which include the locus control region (LCR) [85, 86]. Previous studies have also revealed that LCR has an important of enhancing globin-gene switching potently [86]. Understanding the fetal-to-adult hemoglobin switching is believed to have a clinical relevance of developing novel approaches of reversal of fetal hemoglobin production from adult hemoglobin in patients [87]. Several researchers have applied the knowledge of the regulatory mechanisms of globin-genes expression to design an artificial zinc finger DNA-binding domain (ZF-DBD) with the aim of manipulating the expression patterns of globin-genes [85, 87]. Therefore, techniques involving small molecule inhibitors or genetic knockdown can be potential applications to reactivate the fetal hemoglobin in the future [87].

### 5.4. Posttranslational Regulation of *γ*-Globin Gene Expression

Interestingly, besides the discovery of the upstream signaling pathway of activating the *γ*-globin gene expression, there are findings supporting the fact that posttranscriptional regulation also involves in regulating the *γ*-globin gene expression in response to different stimuli [89, 90]. Weinberg et al. revealed that instead of acting on the transcription rate of *γ*-globin gene in patients, butyrate has the ability to enhance the efficiency of translation of *γ*-globin mRNA [56]. Moreover, there is evidence proving that the key effect of GTP and doxorubicin derivatives is the elevation of the amount of *γ*-globin in patients, at least partially, by enhancing the stability of *γ*-globin mRNA [91, 92]. Liu et al. also suggested that, similar to GTP and doxorubicin derivatives, CuD can therapeutically induce the production of HbF due to its ability of increasing *γ*-globin mRNA stability [1]. They have showed even though the change in the half-life of *γ*-globin mRNA is small, it leads to remarkable changes in the total amount of stable *γ*-globin mRNA and consequently the amount of functional *γ*-globin present in the cell. Therefore, the control of *γ*-globin mRNA stability can be known as a significant regulatory mechanism of *γ*-globin gene expression [93].

## 6. Current Therapeutic Obstacles

In *β*-thalassemia, due to the precipitation of excess α-globin chains, rapid cellular apoptosis of early erythroblasts and red blood cell membrane damage are well characterized [94–96]. 5-Azacytidine, hydroxyurea, myleran, and butyrate had long been applied in clinics for *β*-thalassemia treatment with the aim of stimulating HbF production [97–100]. Despite the ability of inducing the production of HbF in *β*-thalassemia, a large portion of identified HbF-inducing agents, such as 5-azacytidine and hydroxyurea, are chemotherapeutic agents which inhibit cell proliferation and cause cell growth arrest [47]. Further, due to their cytotoxic nature, the dose limiting myelotoxicity and potential carcinogenicity have always led to concerns [76]. Therefore, in order to correct the pathophysiology of *β*-thalassemia, it is of essence to improve the underlying erythroid cell survival and proliferation, with the intention that HbF-inducing agents can exert their effect on stimulating the fetal globin expression prior to the activation of irreversible programmed cell death pathway [47]. Owing to this issue, Perrine et al. (2002) have conducted a pilot study revealing the combination of butyrate and rhu-erythropoietin (EPO), the hematopoietic growth factor that stimulates erythroid proliferation, decreases apoptosis, and prolongs erythroid cell survival and differentiation, has addictive effects in inducing hematologic responses in any *β*-thalassemia patients [101]. This result further suggested that definitive treatment to correct *β*-thalassemia will likely require more than one type of therapeutic regimen; in other words, hematopoietic growth factor, such as exogenous EPO will be required for *β*-thalassemia patients in order to respond optimally to any HbF-inducing agent [47].

Hydroxyurea (HU) was first approved by the Food and Drug Administration (FDA) for the treatment of sickle cell disease (SCD) in 1996 [102]; yet, HU only increases HbF production in approximately half of SCD patients [103] and is even less effective in enhancing the HbF level for *β*-thalassemia patients [104–106]. Additionally, there is a recent clinical trial conducted to assess the therapeutic potential of HQK-1001, an oral butyrate derivative 2,2-dimethylbutyrate sodium salt to treat *β*-thalassemia patients [107]. Although results revealed HQK-1001 exhibits a stimulatory effect on HbF production and *γ*-globin gene expression, further study is needed to find out if the surge of HbF production is sufficient to alleviate the complications of *β*-thalassemia including anaemia, chronic haemolysis, and so forth [107]. Nevertheless, up to date, there is no such pharmacological agent(s) has been officially approved by FDA for treating patients with *β*-thalassemia [102]. The underlying reason of this is that for pharmacological HbF induction therapy in patients with thalassemia major, it requires the production of *γ*-globin chains (plus *β*-globin chains in thalassemia major) reaches 50% of the production of α-globin chains in order to result in optimal therapeutic correction of the anemia in patients with *β*-thalassemia; while for that in patients with SCD, it only requires the production of HbF reaches 20–30% of total circulating hemoglobin for sufficient prevention of sickling effect in SCD [102]. Therefore, it is necessary to identify novel pharmacological HbF inducer or alternate therapeutic approaches which can effectively enhance the HbF production to the optimal level and sufficiently reduce the chain imbalance in homozygous *β*-thalassemia [102].

In recent years, Mabaera et al. (2008) have proposed p38 MAPK cell stress signaling pathway and other stress-related pathways may be the keys to understanding HbF induction, owing to the fact that most HbF-inducing agents are, as mentioned before, cytotoxic and many activate the p38 MAPK cell stress signaling pathway. This stress signaling model predicts a variety of diverse HbF-inducing compounds and stimuli will activate cell stress signaling pathway which will then activate similar response genes, such as the *γ*-globin genes [5]. It is supported by several observation and evidence, for example, the stimulation of *γ*-globin gene expression and HbF production in human primary erythroid cells by 5-azacytidine is closely related to p38 MAPK phosphorylation and this stimulation is inhibited when treated with p38 MAPK inhibitor SB203580 [5]. On the other hand, based on the stress signaling model, Mabaera and colleagues (2008) have also made some predictions which require further investigation, for example, the members of stress signaling pathways, ranging from the sensors of cellular stress to the activated transcription factors that bind to the *γ*-globin gene promoters, are needed for activating the *γ*-globin gene expression as well as HbF production [5].

Nevertheless, concerns have been raised about the possibility of triggering rapid cellular apoptosis in erythroid cells and consequently leading to low blood count when patients, especially those with thalassemia, are receiving chemotherapeutic agents including 5-azacytidine, decitabine, HU and butyrate [5]. Comparing with SCD patients, *β*-thalassemia patients are more susceptible to chemotherapeutic agents because treating with chemotherapeutic agents may further encourage rapid cellular apoptosis which has already been well-characterized in *β*-thalassemia patients even they are not exposed to the agents. Also, as mentioned before, accelerated cellular apoptosis of erythroid progenitors in *β*-thalassemia is a significant barrier to definitive therapy because there is a high possibility that the programmed cell death will be established earlier before HbF-inducing agents act its beneficial effect on the globin chain balance in cells [47]. Consequently, chemotherapeutic agents, such as HU, butyrate and 5-azacytidine, may not be the best HbF-inducer for *β*-thalassemia due to the fact that the HbF-inducing property of those agents are largely dependent on the activation of cell stress signaling pathway. In contrast, those chemotherapeutic agents may have beneficial effect on activating *γ*-globin gene expression and HbF production in SCD; yet, the dose of inducing agents must be strictly monitored in order to ensure it is high enough to activate stress signaling pathway but not too high to trigger cell-cycle arrest or apoptosis in hematopoietic precursor cells that will result in dangerously low blood counts.

## 7. Future Perspectives

In light of the lack of promising HbF-inducing agents for *β*-thalassemia treatment, up to now, different research groups have paid lots of effort on identifying the existing or novel natural Chinese herbal medicines which have the possibility to effectively induce HbF synthesis without any apparent growth-inhibitory effects. YiSui ShenXu Granule (YSSXG), a complex prescription consisting of 11 Chinese herbal medicines, had been used for treating *β*-thalassemia for more than over 20 years [76, 79]. Recently, effort has been put on investigating the efficacy and safety of YSSXG by a randomized single-blinded trial. Result has demonstrated it has obvious clinical efficacy, while hepatauxe and splenomegaly were relieved and no adverse reaction was observed [108]. The underlying mechanism for the effect of YSSXG is possibly by activating the expression of *γ*-globin gene and increase HbF production in order to compensate for the functional deficiency of *β*-globin gene [20]. Zhang and Wu have found out that stimulating the gene expression of *γ*-globin, EpoR, Spi, and FKLF, while hindering the gene expression of Ckit, GATA1, and GATA2, could promote the *γ*-globin gene expression and alter the expression of gene which is responsible for the regulation of *γ*-globin gene expression and the expression of other genes that are participated in the survival, proliferation, and terminal differentiation of erythroid cells [76].

Apart from that, Li et al. (2011) have presented the first piece of evidence on the HbF-inducing property of ethanol extract of* Fructus trichosanthis* (FT), one of the most frequently used Chinese herbal medicine [31]. Their study has demonstrated FT has the ability to enhance the *γ*-globin gene expression as well as HbF synthesis through activating p38 MAPK and inhibiting ERK signaling pathway [31]. Despite the promising result shown, acute and chronic toxicity test* in vivo* is strongly required in the future in order to ensure the ethanol extract of FT is safe for clinical use on human. Also, the efficacy of the extract should be examined in clinical evaluation before actual clinical practice [31]. During the same year, another group of researchers have identified Cucurbitacin D (CuD), a chemical inducing agent that can be found in* Fructus trichosanthis*, is a novel therapeutic agent for treating *β*-thalassemia [1]. There are evidences suggesting that CuD could act as a good HbF-inducing agent for *β*-thalassemia patient comparing with HU since CuD exhibits a higher amplitude and rapidity in enhancing the HbF production than HU does in K562 cells, and, unlike HU, CuD does not show any growth-inhibitory effect even when it is at its optimal activity [1]. Taken all together, natural herbal medicines, which exhibit a higher potency in HbF induction compared with chemotherapeutic agents and have much lower cytotoxicity, will definitely be the novel therapeutic candidates for *β*-thalassemia by targeting the activation of *γ*-globin gene expression; yet, some of the candidates are still required further investigation on their safety and efficacy [1].

## 8. Conclusion

Chemotherapeutic agents, such as 5-azacytidine, hydroxyurea, myleran, and butyrate, had long been used for *β*-thalassemia treatment by stimulating HbF synthesis; yet, cytotoxicity, growth-inhibitory effect, fear of long-term carcinogenesis, and only modest HbF-inducing activity have limited the clinical usage of these agents in *β*-thalassemia and SCD treatment. Also, through understanding the pathology of *β*-thalassemia, it is revealed that most of the identified HbF-inducing agents have limitation on treating *β*-thalassemia. It is because the rapid cellular apoptosis of erythroid progenitors in *β*-thalassemia causes a significant obstacle that overstimulating the cell stress signaling pathway by the HbF inducer may possibly lead to irreversible cellular apoptosis before *γ*-globin gene expression and HbF synthesis can be stimulated. With the advancement of biotechnologies, increasing number of studies will be done to explore and optimize new interventions and nature remedies to reactivate HbF synthesis for *β*-thalassemia patients. In the future, it is expected that increasing number of HbF inducing agents could be found from natural remedies and folk medicines all over the world. In this context, further studies are required with the aim of exploring more natural herbal medicines as well as studying the efficacy and safety of from the laboratory to clinical use for the individuals with *β*-hemoglobinopathies.

## Figures and Tables

**Table 1:** Classification of HbF inducers.

Category	Examples of Inducers	Mechanism of action
Chemotherapeutic agents (ribonucleotide reductase inhibitors)	HU	Inhibition of DNA analysis [13, 47]

Chemotherapeutic agents (DNA methyltransferase inhibitors)	5-Azacytidine, decitabine and citarabine	Hypomethylation of DNA [13, 47]

Short chain fatty acids and derivatives (histone deacetylase inhibitors)	Butyrates, tricostatin, apicidine, and scriptaid	Inhibition of histone deacetylase (HDAC) activity (applicable to some SCFAs only) [47, 56–65]

DNA binding agents	Mithramycin, cisplatin and analogues, tallimustine and analogues, and angelicin	DNA-binding activity [5, 50]

mTOR inhibitors	Rapamycin	FRAP-mTOR signal transduction targeting [49]

Immunomodulatory drugs	Thalidomide, revlimid, and Pomalidomide	Histone acetylation at *γ*-globin gene promoter [5, 66]

Cytokines	Erythropoietin (EPO), stem cell factor and TGF-*β*	Increase in the frequency of erythroid progenitors programmed to hemoglobin F [5, 47, 52, 53, 67, 68]

**Table 2:** Natural remedies as HbF inducer.

Inducer	Source	Biological effects
Bergaptene	*Aegle marmelos *	Erythroid differentiation of K562 cells, HbF induction [31, 72]

Angelicin	*Angelica arcangelica *	Erythroid differentiation, HbF induction [8, 73]

Resveratrol	Red wine, grape skin, and darakchasava	HbF induction [8, 74]

Citropten and bergapten	Bergamot orange	Erythroid differentiation and HbF production [69]

Rapamycin	*Streptomyces hygroscopicus *	HbF induction [8, 13]

Mithramycin	*Streptomyces *species	HbF production [75]

YiSui ShengXue Granule		Erythroid survival, proliferation, and terminal differentiation of K562 cells and HbF production [76]

Cucurbitacin D	Ethanol Extract of *Fructus trichosanthis, *which is the fruit of *Trichosanthes kirilowii *MAXIM	HbF production and Erythroid differentiation of K562 cells [1, 31]

Niprisan/Nix-0699/Nicosan	A ethanol/water extract developed in Nigeria from indigenous plants	Anti-sickling effect [31, 70, 71]

*Terminalia catappa* distilled water active fraction (TCDWF)	*Terminalia catappa* leaves	Erythropoiesis, cell proliferation, and transcription [77]
